# IL-27 producers in a neonatal BCG vaccination model are a heterogenous population of myeloid cells that are diverse in phenotype and function

**DOI:** 10.1093/immhor/vlaf003

**Published:** 2025-03-06

**Authors:** Ashley M Divens, Li Ma, Jordan K Vance, Jessica M Povroznik, Gangqing Hu, Cory M Robinson

**Affiliations:** Department of Microbiology, Immunology, & Cell Biology, West Virginia University School of Medicine, Morgantown, WV, United States; Department of Microbiology, Immunology, & Cell Biology, West Virginia University School of Medicine, Morgantown, WV, United States; Department of Microbiology, Immunology, & Cell Biology, West Virginia University School of Medicine, Morgantown, WV, United States; Department of Microbiology, Immunology, & Cell Biology, West Virginia University School of Medicine, Morgantown, WV, United States; Department of Microbiology, Immunology, & Cell Biology, West Virginia University School of Medicine, Morgantown, WV, United States; Department of Microbiology, Immunology, & Cell Biology, West Virginia University School of Medicine, Morgantown, WV, United States; Vaccine Development Center, West Virginia University Health Sciences Center, Morgantown, WV, United States

**Keywords:** BCG, interleukin-27, neonate, tuberculosis, vaccine

## Abstract

Tuberculosis (TB) is a serious public health concern in many regions of the world and the only approved vaccine to prevent TB is the live-attenuated BCG vaccine. Despite being widely used, the BCG vaccine fails to prevent pulmonary TB in adults. The BCG vaccine is administered during the neonatal period when levels of the immunosuppressive cytokine interleukin (IL)-27 are elevated, and previous studies have demonstrated that the source of IL-27 can impact downstream immune responses. We therefore sought to characterize the specific subpopulations of myeloid cells that produce IL-27 following BCG vaccination. To investigate this, we administered the BCG vaccine to neonatal IL-27p28eGFP mice that report IL-27 production. Our studies demonstrated that BCG vaccination steadily increased IL-27 production throughout the weeks post-vaccination. We also showed that a predominantly CD11b^+^ F4/80^+^ population of IL-27 producers increased MHC class II expression following BCG vaccination in both the spleen and the lung. However, producers of IL-27 in these tissues differ, with a population of CD11c^+^ MHC II^+^ cells emerging in the spleen and a subset of Ly6G/C^+^ MHC II^+^ emerging in the lung. 10x scMultiome analysis further validated the increase in MHC class II expression and demonstrated improved antigen presentation functionality following vaccination. The sequencing analysis also revealed subpopulations of IL-27 producers with immunosuppressive functions such as a population of macrophages with increased *Mrc1* expression post-vaccination. Our findings suggest that IL-27 producers are a heterogenous population of myeloid cells that impact the development of protective immune responses induced by the BCG vaccine.

## Introduction

Tuberculosis (TB) is a serious public health concern in many regions of the world, including parts of Asia, South America, and Africa.^1^ Currently, the bacille Calmette-Guérin (BCG) vaccine is the only vaccine approved to prevent TB, and it is most commonly administered in regions of the world where TB is endemic.^1^ The BCG vaccine is a live-attenuated strain of *Mycobacterium bovis* that was developed by repetitive subculturing of a virulent strain until it failed to cause disease in animal models.^2^ This vaccine has been administered more than 4 billion times, making it one of the most widely used vaccines in the world.^3^^,^^4^ Despite being widely used, the BCG vaccine has variable protective efficacy against TB. The vaccine is effective in preventing disseminated TB in children but fails to confer long-term protection against pulmonary TB in adults.^4–6^ The 1.3 million deaths caused by TB in 2022 highlights the need for improved vaccination strategies.^1^

A variety of hypotheses have been proposed to describe the inadequacies of the BCG vaccine. A popular hypothesis is that prior sensitization by environmental mycobacteria can prevent BCG replication and block further protection induced by the BCG vaccine.^4^^,^^7–9^ Other factors that may be contributing to the inefficacy of the BCG vaccine include route of administration, time of administration, and batch variations.^8^^,^^10^ Another reason the BCG vaccine may fail to be protective long-term is increased production of the immunosuppressive cytokine interleukin (IL)-27 at the time of vaccination. Administration of the BCG vaccine occurs during the neonatal period, defined as the first 28 days of life in humans.^1^^,^^11–13^ Studies have shown that IL-27 is elevated during the neonatal period by comparing gene expression and secreted cytokine in human macrophages from cord blood versus adult peripheral blood.^14^^,^^15^ The increase of IL-27 during the neonatal period is also observed in mice with increased IL-27 expression in the spleen and serum compared to adults.^14^^,^^16^

IL-27 is a heterodimeric, cytokine comprised of the IL-27p28 and EBI3 subunits.^17^ It is a member of the IL-12 family of cytokines that also includes IL-12, IL-23, and IL-35.^18^ IL-27 is primarily produced by myeloid cells including macrophages, dendritic cells, and myeloid-derived suppressor cells.^16^^,^^19^^,^^20^ However, there is also evidence that suggests B cells, plasma cells, and endothelial cells may be sources of IL-27.^19^^,^^21–24^ IL-27 signals through a receptor comprised of IL-27Rα and gp-130 that is expressed broadly by both immune and non-immune cells.^19^^,^^25^^,^^26^ IL-27 was first identified for its ability to induce Th1 responses, such as the production of IFN-γ from CD4^+^ T cells.^17^^,^^27^ However, later studies demonstrated immune suppressive roles for IL-27 including the inhibition of T cell responses, the production of IL-10 by regulatory T cells, compromised control of bacteria by innate immune cells, and reduced lysosomal trafficking.^14^^,^^19^^,^^28–32^ Overall, IL-27 is a pleiotropic cytokine that is expressed by a variety of cells and promotes diverse outcomes under different conditions.

Our laboratory has previously shown that IL-27 is further elevated following stimulation of macrophages and dendritic cells with BCG in vitro or vaccination of neonatal mice with BCG.^14^^,^^29^^,^^32^ Furthermore, this increase in IL-27 interferes with the phagolysosomal pathway and inhibits efficient clearance of BCG.^29^^,^^32^ Mycobacterial persistence favors a sustained effector memory T cell response over the development of a central memory response, and we further predict that inadequate lysosomal processing may decrease the diversity of antigens presented to T cells.^4^^,^^33^^,^^34^ In a prior study, we demonstrated that in the absence of IL-27 signaling BCG-vaccinated mice exhibit superior clearance of *M. tuberculosis* (Mtb) with a more multifunctional cytokine response in the lungs following challenge.^35^ Collectively, these ideas point to important implications for IL-27 in neonatal vaccination and generation of protective immunity.

The specific cellular producers of IL-27 following BCG vaccination have not yet been elucidated. Since the cellular source of IL-27 can impact downstream signaling and subsequent vaccine responses,^36^ this study sought to determine the specific subpopulations of cells that produce IL-27 and gain insight into their functionality following BCG vaccination. The following studies were completed using a murine neonatal BCG vaccination model that we previously developed.^29^^,^^35^ IL-27 production following vaccination was evaluated using IL-27p28eGFP reporter mice that express GFP under control of the IL-27 regulon, and were vaccinated as neonates.^36^ These experiments demonstrated increased GFP expression, indicative of IL-27 production, in the spleen and lung in the weeks following BCG vaccination, with different kinetics for each tissue. Surprisingly, IL-27 production did not increase immediately following vaccination and instead begins to increase around 3 weeks post-vaccination, with a continual increase throughout 5 weeks post-vaccination. Contrary to our hypothesis, persistent BCG was not responsible for this continual increase in IL-27. IL-27-producers expressed a variety of myeloid markers, with most producers F4/80^+^ and CD11b^+^ in both the spleen and the lung. Notably, the level of MHC class II expression increased following vaccination, suggestive of improved antigen presentation. Simultaneous analysis of gene expression and chromatin accessibility at single cell levels through 10X scMultiome complemented the phenotypic analysis of IL-27 producers and identified unique populations of cells with both inflammatory and immune-suppressive functions post-vaccination. To our knowledge, this is the first report to describe the cellular sources of IL-27 and their corresponding transcriptomic and epigenomic profiles following BCG vaccination.

## Methods

### Animals

C57BL/6J mice were purchased from the Jackson Laboratory (Bar Harbor, Maine, USA). IL27p28eGFP mice on a C57BL/6J background were a kind gift from Dr Ross Kedl at the University of Colorado School of Medicine.^36^ Breeding pairs were maintained at West Virginia University School of Medicine. Litters had an average of 7 pups with approximate equal distribution of males and females and both sexes were used for all experiments. The neonatal period for all experiments was defined as the first 8 days of life, as described by previous literature.^13^^,^^14^^,^^16^^,^^29^^,^^35^ All procedures were approved by the West Virginia University Institutional Animal Care and Use Committees.

### *Mycobacterium bovis* BCG culture

*Mycobacterium bovis* BCG strain TMC 1011 (Pasteur) was purchased from American Type Culture Collection (ATCC; Manassas, Virginia, USA). Liquid cultures were started from a single colony on Middlebrook 7H10 agar (Difco; Sparks, Maryland, USA) and grown with shaking at 37°C in Middlebrook 7H9 broth base (HiMedia; Mumbai, India) supplemented with 10% OADC (oleic acid, albumin, dextrose, and catalase) and 14.4 M pimaricin (Sigma; St Louis, Missouri, USA). To prepare frozen stocks, cultures were then pelleted by centrifugation at 2,000 × *g* for 5 minutes and resuspended in Middlebrook 7H9 broth base with 30% glycerol. The cultures were passed through a 27-gauge needle to disrupt clumps prior to distributing into separate frozen vials. Titers were determined after 1 freeze-thaw cycle.

### BCG inoculum and neonatal vaccination

Pre-titered frozen BCG cultures were thawed, vortexed, and passed through a 27-gauge needle to disrupt clumps. The BCG was then washed with phosphate-buffered saline (PBS) and centrifuged to remove the freezing media. Following centrifugation, BCG were sonicated in a water bath and again passed through a 27-gauge needle. The inoculum was resuspended in PBS to achieve a final volume of 50 µl per mouse and titered to determine the actual dose. Mice were vaccinated in the subscapular region during the neonatal period at 7 days of life with a target dose of 10^3^ CFUs/mouse. Control mice were administered 50 µl of PBS in parallel with the vaccinated mice. In some experiments streptomycin sulfate (Sigma-Aldrich, St Louis, Missouri. USA) dissolved in 0.9% isotonic saline was intraperitoneally injected (150 mg/kg) beginning 1-week following BCG vaccination and maintained every other day for 2 weeks.

### Flow cytometry

At varied timepoints post BCG vaccination, mice were humanely euthanized, and the spleens and lungs were harvested for immunolabeling. Spleens were crushed through 40 µm mesh strainers using DMEM (Cytiva; Logan, Utah, USA) to create single cell suspensions and then pelleted by centrifugation at 350 ×*g* for 5 minutes. RBCs were subsequently lysed using ACK lysis buffer (Gibco; Grand Island, New York, USA) for 5 minutes, and the lysis buffer was neutralized using DMEM supplemented with 10% FBS. The splenocytes were pelleted again and resuspended in buffer (1x PBS, 0.5% BSA, and 2 mM EDTA) for counting. Lungs were dissociated to create single cell suspensions using the mouse lung dissociation kit (Miltenyi Biotec; Bergisch Gladbach, Germany) and the gentleMACs Octo Dissociator (Miltenyi Biotec). The cells were then pelleted by centrifugation at 350 × *g* for 5 minutes. RBCs were lysed as described above. The cells were pelleted again and then pushed through a 40 µm mesh strainer to ensure the creation of a single cell suspension. The cells were then resuspended in buffer for counting. All cells were mixed with 10 µls of FcR Blocking Reagent (Miltenyi Biotec) per 10^7^ cells for 10 minutes at 4°C. Cells were labeled for 45 minutes at 4°C with 1:1,500 dilution of fixable viability dye 780 (APC-Cy7) and the following antibodies (BD Biosciences; Franklin Lakes, NJ):; 0.125 µg of Ly6G/C (APC), F4/80 (PE), CD45 (R718), CD11b (BV786); 0.25 µg of CD11c (BV510), MHCII (BV650), CD3 (PE-CF594), CD19 (PE-CF594); and 0.5 µg of CD204 (BB700). Cells were washed and fixed with 0.4% paraformaldehyde overnight at 4°C. A minimum of 50,000 events were collected using the BD LSR Fortessa (BD Biosciences; Franklin Lakes, New Jersey, USA). Results were analyzed using FCS Express 7 Research Edition (De Novo Software; Pasadena, California, USA).

### Cytokine detection

Blood collected from unvaccinated or BCG vaccinated mice by submandibular bleeding was centrifuged for 10 minutes at 2,000 × *g* to collect serum that was stored frozen at −80°C until use. The serum IL-27p28 levels were measured using the V-PLEX Plus Mouse IL-27p28/IL-30 Kit electrochemiluminescence assay (Meso Scale Discovery; Rockville, Maryland, USA) according to manufacturer instructions. The serum samples were thawed and diluted 4-fold, per protocol recommendations. The results were analyzed using the Discovery Workbench desktop analysis software version 4 (Meso Scale Discovery).

### BCG enumeration

To enumerate BCG persistence in peripheral tissues, spleens were homogenized in 250 µl of PBS using a handheld homogenizer, while lungs and livers were homogenized in 500 µl of PBS. BCG in the homogenized tissues was enumerated by standard plate counts on Middlebrook 7H10 agar supplemented with OADC and pimaricin. The plates were incubated for 14 days at 37°C and then counted to determine CFUs/tissue.

### Nuclei isolation from GFP^±^ cells

At 5 weeks post-vaccination, spleens were harvested from IL-27p28eGFP BCG-vaccinated mice. The spleens were then dissociated to a single cell suspension and prepared for immunolabeling as described above. Cells were labeled for 45 min at 4°C with 1:1500 dilution of fixable viability dye 780 (APC-Cy7) and the following antibodies (BD Biosciences; Franklin Lakes, New Jersey, USA): 0.25 µg of CD3 (PE-CF594) and CD19 (PE-CF594). Live GFP^+^ CD3/CD19^-^ cells were sorted with a FACSAria III Cell Sorter, collected into 10% BSA, and counted. Nuclei were then isolated from the GFP^+^ cells according to the 10X Nuclei Isolation for Single Cell Multiome ATAC + Gene Expression Sequencing protocol (CG000365). Briefly, the cells were centrifuged at 300 × *g* for 5 minutes at 4°C. Lysis buffer was then added to the cell pellet and incubated for 5 minutes. The cells were then washed and centrifuged at 500 × *g* for 5 minutes at 4° 3 times. The cells were then resuspended in chilled diluted nuclei buffer, and the nuclei concentration was determined.

### Transposition, GEM generation & barcoding, and library pre-amplification PCR

Transposition, GEM generation and barcoding, and library pre-amplification was performed as recommended by 10x Genomics (Pleasanton, California, USA) using a Chromium Single Cell Multiome ATAC + Gene Expression kit. Briefly, isolated nuclei were diluted and incubated with a transposition mix (ATAC Buffer B and ATAC Enzyme B) to fragment the DNA and add adapter sequences. The transposed nuclei were then combined with the master mix (barcoding reagent mix, template switch oligo, reducing agent B, and barcoding enzyme mix), gel beads, and partitioning oil in the 10x Chromium Next GEM Chip J to produce GEMs. Each sample was then amplified via PCR to be used for ATAC library construction and cDNA amplification for gene expression library construction.

### Single cell multiome (scMultiome) library construction

Libraries were prepared as previously described and as recommended by 10x Genomics using a Chromium Single Cell Multiome ATAC + Gene Expression kit.^37^

### Single cell multiome data analysis

Sequencing reads were aligned to the GRCm38 (mm10) reference genome. ATAC and GEX molecules were quantified using cellranger-arc count (version 2.0.1). Seurat (version 5.0.1) and Signac (1.13.0) were used for downstream analysis.^38^^,^^39^ All samples’ data were combined into a merged Seurat object. For the merged object, the mitochondrial and ribosomal genes from the RNA gene expression matrix was filtered out for downstream analysis.

For RNA data, NormalizeData was used for normalization and the normalization method was set as “CLR” (centered log ratio transformation). Cell cycle phase score was calculated by CellCycleScoring. Variable features were identified by FindVariableFeatures and the top 2,000 features were selected. Data were scaled and centered by ScaleData function and regress out the cell cycle’s effect. RunPCA was used for principal component analysis (PCA) dimensionality reduction. RunUMAP was used for dimensional reduction and visualization via uniform manifold approximation and projection (UMAP). FindNeighbours was used to compute the nearest neighbors for the object. FindClusters was used to identify clusters of cells by a shared nearest neighbors modularity optimization based on original Louvain clustering algorithms.

For ATAC data, RunTFIDF was used to compute term-frequency inverse-document frequency normalization on the matrix. FindTopFeatures was used to find the most frequently observed features. RunSVD was used to find the top 50 largest singular value and corresponding singular vectors of the matrix. RunHarmony was used to correct batch effects.^40^ RunUMAP was used for dimensional reduction and visualization via uniform manifold approximation and projection (UMAP). FindNeighbours was used to compute the nearest neighbors for the object. FindClusters was used to identify clusters of cells by a shared nearest neighbors’ modularity optimization based on Smart Local Moving (SLM) clustering algorithms.^41^

Weighted nearest neighbor (WNN) analysis was conducted to integrate the RNA and ATAC data. FindMultiModalNeighbors was used to construct the WNN graph and it was based on a weighted combination of the RNA and ATAC data. RunUMAP was used for dimensional reduction and visualization via uniform manifold approximation and projection (UMAP). FindNeighbours was used to compute the WNN for the object.

Each RNA, ATAC and WNN subclusters’ features were plotted to confirm their quality. After filtering out the subclusters with significantly low levels of RNA fragments and/or ATAC fragments, further quality control was conducted with the following settings: nCount_ATAC (number of fragments) > 1,000, nCount_ATAC < 40,000, nCount_RNA (total number of RNA fragments) > 100, nFeature_ATAC (number of peaks) > 500, nFeature_ATAC < 20,000, nFeature_RNA (number of genes) > 50, nucleosome signal < 1, pct_reads_in_peaks > 40%, percent.mt < 60%, percent.mt.atac < 1%, TSS.enrichment > 2 and blacklist_fraction < 5%. Percent.mt (RNA reads from mitochondrial genes) was calculated by the PercentageFeatureSet function. Percent.mt.atac was calculated by 100*atac_mitochondrial_reads/atac_raw_reads.

After the above quality control procedures, the effect of cell cycle, RNA and ATAC sequencing depth, and mitochondrial and ribosomal fragments were regressed out using ScaleData. After getting the dimensional reduction results of RNA and ATAC, RunHarmony was used to correct their batch effects, respectively.^40^

Following the same procedure described above, RNA, ATAC and WNN subclusters was identified use FindClusters. Small subclusters with significantly low levels of RNA fragments and/or ATAC fragments were filtered out for downstream analysis.

### Cell type identification

FindAllMarkers was used to identify the marker genes for each subcluster, as compared to the other clusters, by setting “wilcox” (Wilcoxon Rank Sum test) as the test used, min.pct as 0.1, and logfc.threshold (fold change in log2 scale) as .25. Cell types were identified based on the subclusters’ marker genes, and their enrichment analysis from the Metascape enrichment analysis. Seven cell types were identified: monocytes (Cebpb, Cybb, Itgal), monocyte-derived dendritic cells (MoDC) (Ccr2, Ly6c2, H2-Ab1), macrophages (C1qa, Vcam, Mrc1), population 1 of conventional dendritic cells (cDC-1) (Relb, Traf1, Ccr7), population 2 of conventional dendritic cells (cDC-2) (Cd74, H2-Ab1, Ciita), plasmacytoid dendritic cells (pDC) (Tcf4, Grm8, Prkca), and neutrophils (Lcn2, Camp, Ltf). The two populations of conventional dendritic cells share many similarities but exhibit two distinct ATAC subclusters.

### Inferred gene activity from single cell ATAC data

The GeneActivity function in Signac (version 1.13.0) was used to quantify the activity of each gene in the genome by assessing the chromatin accessibility associated with the gene. Extend.upstream (number of bases to extend upstream of the transcription start site) was set as 3,000. Extend.downstream (number of based to extend downstream of the transcription termination site) was set as 0.

### Gene ontology (GO) and pathway enrichment analysis

Metascape was used for GO and pathway enrichment analysis with the complete Mus musculus proteome as the background.^42^ The Metascape output provided a list of the top 20 enriched items for each cell type's up-regulated genes when comparing the treatment to the control. From this, we selected specific items for each cell type and integrated them to visualize their significance and enrichment across different cell types.

### Differentially expressed genes (DEGs) between treatment and control

Each cell type was extracted to identify cell type specific differentially expressed genes (DEGs) between treatment and control. FindMarkers was used to identify the DEGs for the comparison, by setting “wilcox” (Wilcoxon Rank Sum test) as the test used, min.pct as 0.1, and logfc.threshold (fold change in log2 scale) as 0.25. Genes with a *P*-value below 0.01 were considered significant.

### Data accessibility

All raw sequenced data are publicly available at Gene Expression Omnibus under accession number GSE281538. Secure token for data access during manuscript peer-review is alwruekunlwrnsb. https://www.ncbi.nlm.nih.gov/geo/query/acc.cgi?acc=GSE281538

### Statistical analysis

All statistics were performed using GraphPad Prism Version 9.1.1 (San Diego, California, USA). The threshold for statistical significance was set to α = 0.05. The details of analyses for specific data sets are described in the figure legends.

## Results

### GFP expression increases throughout the first five weeks following vaccination in the spleen and lung

**Figure 1.**
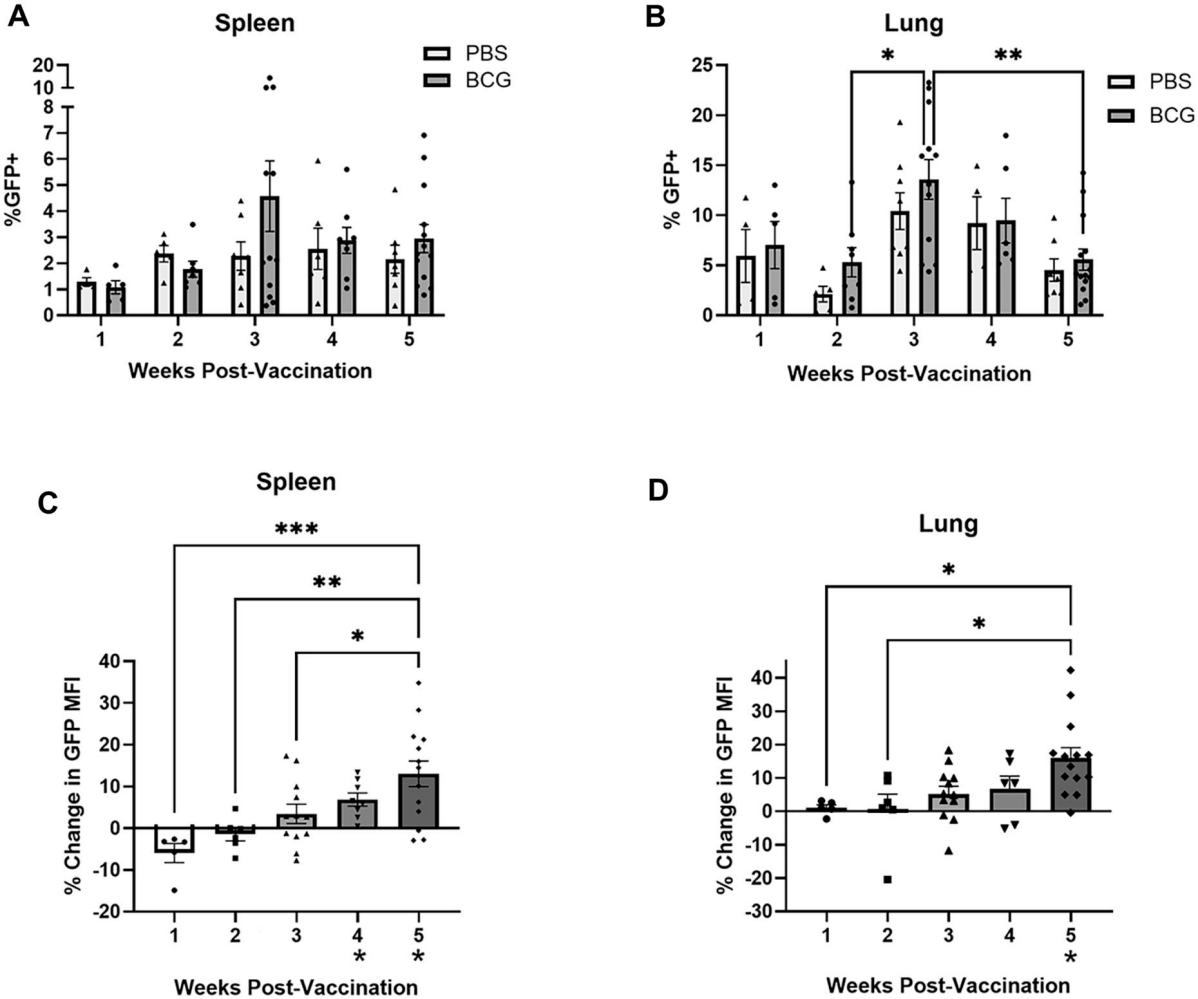
GFP expression increases throughout the first 5 weeks following vaccination in the spleen and lung. At different weeks post-vaccination (WPV), spleens and lungs were collected from GFP^+^ mice. Splenocytes and lung cells were labeled with a live/dead stain and CD3/CD19 antibodies to remove the lymphocyte population. Flow cytometry was performed on the labeled cells. Data is reported as (A, B) the mean percent of GFP^+^ cells ± SE and as (C, D) the mean percent change of the median fluorescent intensity (MFI) of GFP expression ± SE for BCG vaccinated mice relative to PBS controls across timepoints. Asterisks on the *x*-axis (C, D) indicate significance compared to PBS controls. Data in the 1 WPV timepoint are representative of 2 independent experiments and data in the rest of the timepoints are representative of at least 3 independent experiments. *Statistical analysis*: (A, B) Two-way ANOVA, followed by Tukey’s multiple comparisons test, (C, D) One-way ANOVA (across timepoints) and unpaired t test with Welch’s correction (compared to PBS controls); *P < 0.05, **P < 0.01, ***P < 0.001.

Previous studies from our laboratory demonstrated that there is a continuous increase in IL-27 serum levels throughout the weeks following BCG vaccination.^29^ In line with this observation, we sought to determine the source and level of IL-27 expression in the weeks following BCG vaccination in both the spleen and the lung. The spleen represents an important and representative secondary lymphoid organ, whereas the lung is a target tissue implicated in control of *Mycobacterium tuberculosis* infection. To address this, we used an IL-27p28eGFP (GFP) reporter mouse that has been shown to reliably report IL-27 expression.^36^ GFP mice were vaccinated according to our previously described neonatal vaccination model to match the time of life and stage of immunity that BCG vaccination occurs in humans.^29^ Spleens and lungs were harvested at varied timepoints post-vaccination, and the tissues were labeled using a live/dead stain and CD3/CD19 antibodies for flow cytometry analysis. T and B cells were removed from the analysis as shown in Fig. S1; although some T and B cells have been shown to produce IL-27 in a context-dependent manner, there is no evidence in our model that they do so in response to BCG (data not shown).^22–24^^,^^43^^,^^44^ In both the spleen and lung, the percent of GFP^+^ cells elevated at various timepoints in the BCG vaccinated mice compared to unvaccinated mice at the same time point, although this does not reach statistical significance (Fig. 1A and B). The percent of GFP^+^ cells in the spleen gradually increased throughout the weeks following vaccination and peaked at 3 weeks post-vaccination (WPV) (Fig. 1A), whereas the percent of GFP^+^ cells in the lung was significantly elevated at 3 WPV compared to 2 and 5 WPV (Fig. 1B). The unvaccinated mice also experienced varied GFP expression across different timepoints, but this is not unexpected as IL-27 gene expression increased in the spleens of naïve mice through 3 weeks of life.^14^ Median fluorescent intensity (MFI), an indicator of the abundance of protein expression, demonstrated that vaccinated animals produce significantly more GFP per cell than unvaccinated controls in the spleen at 4 and 5 WPV (Fig. 1C). A similar pattern was observed in the lung and reached significance at 5 WPV (Fig. 1D). These findings demonstrate that GFP expression rises in the 5 weeks following BCG vaccination in both the spleen and lung, but this increase unexpectedly requires several weeks.

### Circulating IL-27 levels increase throughout the first five weeks following vaccination and correlate with the abundance of GFP in the spleen

**Figure 2.**
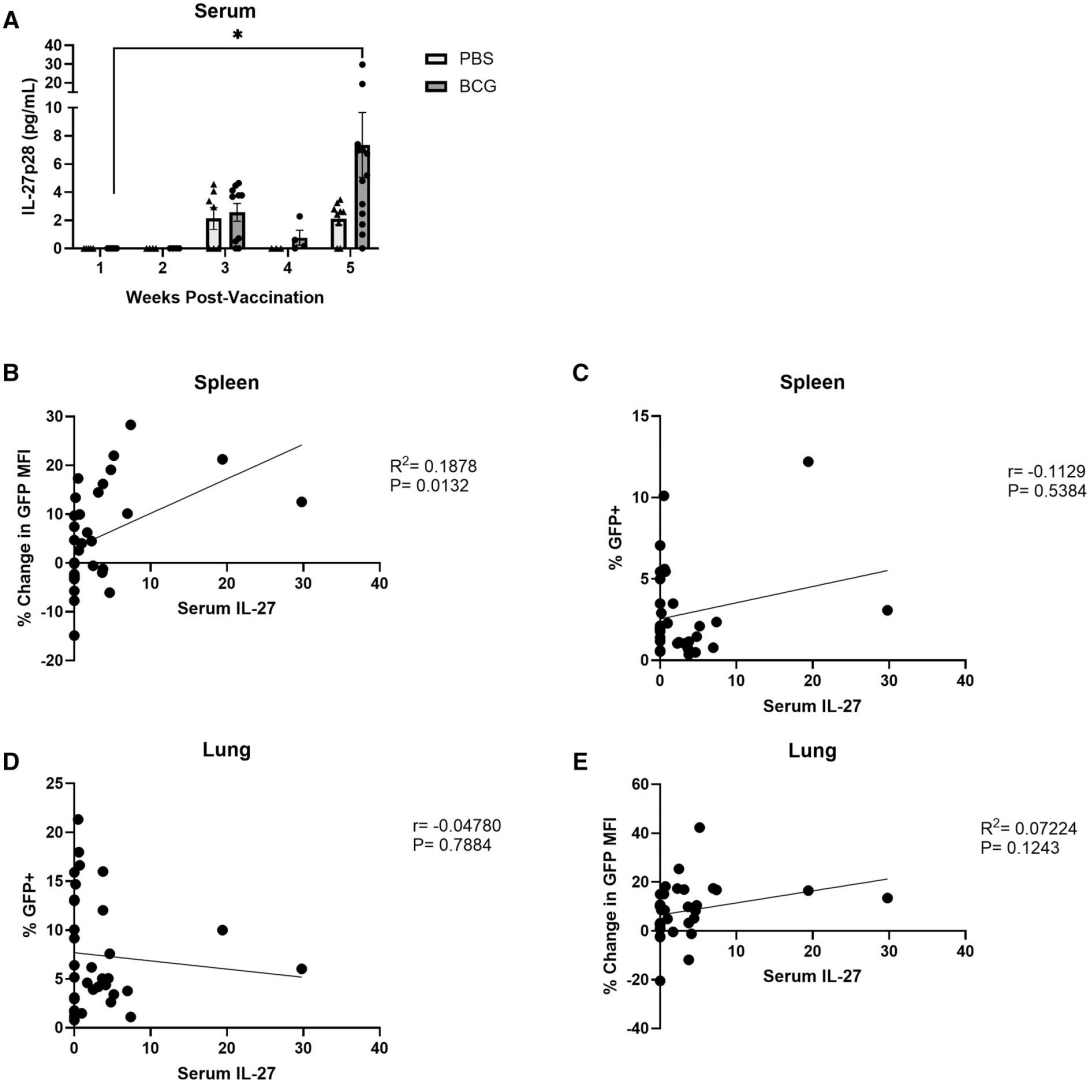
Circulating IL-27 levels increase throughout the first five weeks following vaccination and correlate with the abundance of GFP in the spleen. (A) Serum was analyzed for IL-27 production by electrochemiluminescent detection. Correlation analyses were performed between serum IL-27 levels across all timepoints and the (B) percent of GFP^+^ cells in the spleen, (C) percent change in GFP MFI in the spleen, the (D) percent of GFP^+^ cells in the lung, and (E) percent change in GFP MFI in the lung. Data in the 1 WPV timepoint are representative of 2 independent experiments and data in the rest of the timepoints are representative of at least 3 independent experiments. Statistical analysis: (A) One-way ANOVA among BCG samples, followed by Kruskal-Wallis Test. *P < 0.05; (B, D) Spearman r correlation test; (C, E) Pearson *r* correlation test.

To address if increased IL-27 production in the spleen and lung following BCG vaccination was consistent with circulating cytokine, blood was collected in parallel with the experiments shown in Fig. 1 to evaluate serum IL-27 levels. In agreement with previous results,^29^ BCG vaccination increases serum IL-27 levels in the same animals evaluated for GFP production from 1 through 5 WPV compared to the unvaccinated controls (Fig. 2A). In the spleen the percentage of GFP^+^ cells does not correlate with serum IL-27 levels across all timepoints (Fig. 2B), but there is a significant correlation with the GFP MFI (Fig. 2C). Serum IL-27 levels and GFP expression in the lung did not correlate across all timepoints (Fig. 2D and E), likely because the abundance of producers peaked significantly at 3 weeks post-vaccination. These findings suggest that the total amount of protein made in the spleen contributes significantly to circulating IL-27 levels. Furthermore, these data highlight the spleen as a tissue of interest for further analysis.

### The frequency of IL-27 producers in the spleen that express MHC class II and CD11c increases following BCG vaccination

**Figure 3.**
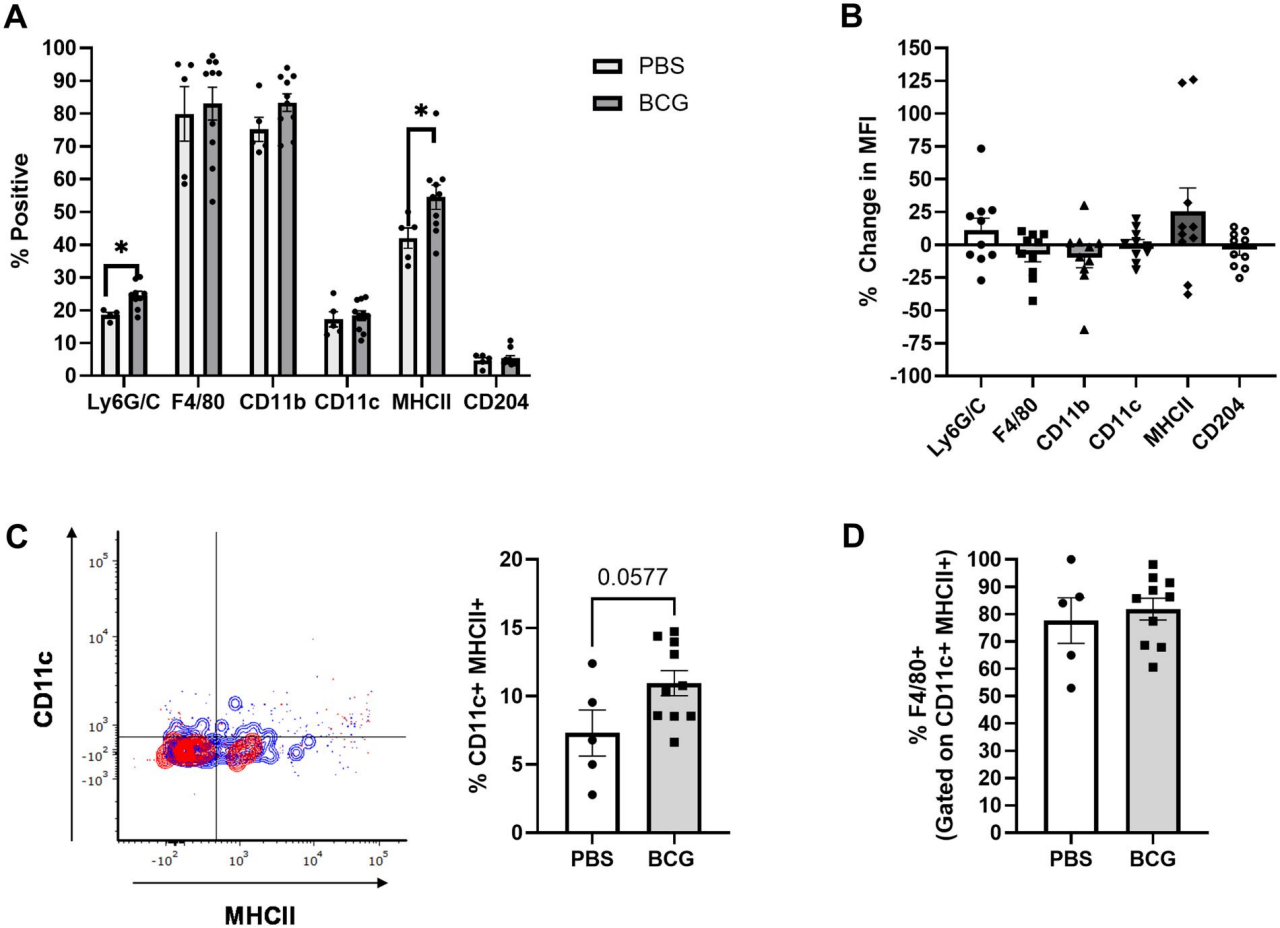
The frequency of IL-27 producers in the spleen that express MHC class II and CD11c increases following BCG vaccination. At 5-weeks post-vaccination (WPV), spleens were collected from GFP mice. The splenocytes were labeled with a live/dead stain and antibodies for CD3/CD19, CD45, Ly6G/C, F4/80, CD11b, CD11c, MHC II, and CD204. Flow cytometry was performed on the labeled cells. All data shown is gated on live CD3/CD19^-^GFP^+^ CD45^+^ cells. Data for individual markers is reported as (A) the mean percent of GFP^+^ cells ± SE also positive for the indicated markers and as (B) the mean percent change of median fluorescent intensity (MFI) of each marker between the PBS controls and BCG vaccinated mice ± SE. (C) A representative flow plot (red = control, blue = BCG vaccinated) and corresponding mean CD11c^+^MHCII^+^ percent of total cells ± SE is shown for both the control and BCG vaccinated groups. Data are representative of 4 independent experiments. (D) The mean ± SE total frequency of CD11c^+^MHCII^+^ cells that express F4/80 is also shown. Statistical analysis: (A, C, D) Unpaired *t* test, (B) Brown Forsythe and Welch ANOVA tests; *P < 0.05.

In addition to understanding the kinetics and abundance of IL-27 producers throughout the weeks following BCG vaccination, we also wanted to understand the cellular phenotypes. It was important to determine the specific subpopulations that produce IL-27 following BCG vaccination, as prior studies have shown the cellular source of IL-27 under different conditions can dictate T cell responses.^19^^,^^36^^,^^45^ Since the abundance and magnitude of IL-27 production was highest at 5 weeks post-vaccination (WPV) in the spleen, we focused on this timepoint. Previous literature has established that the dominant producers of IL-27 are myeloid cell populations including macrophages, dendritic cells, and myeloid-derived suppressor cells, and therefore we developed a myeloid panel to allow us to phenotypically profile IL-27 producers via flow cytometry.^16^^,^^19^^,^^20^ Spleens were harvested at 5 WPV and immunolabeled for flow cytometry analysis, with each myeloid panel marker gated on live CD3/CD19^-^ GFP^+^ CD45^+^ cells (Fig. S1). Analysis of the total percent of GFP^+^ cells that expressed individual myeloid cell markers demonstrated that most IL-27 producers were F4/80^+^ and CD11b^+^ with some differences between the control and vaccinated groups, particularly in the frequency of Ly6G/C and MHC class II (MHC II) expression (Fig. 3A). The percent change in MFI of the indicated markers confirmed that cells increased their MHC II expression level following BCG vaccination relative to unvaccinated animals (Fig. 3B). The increased frequency of GFP^+^ MHCII^+^ cells post-vaccination suggests these cells may have improved antigen presentation capabilities. To further determine the subpopulation of cells producing IL-27, we analyzed the co-expression of multiple markers. There was a nearly significant increase in the percent of GFP^+^ cells that are CD11c^+^ MHCII^+^ following vaccination (Fig. 3C). CD11c is typically viewed as a canonical dendritic cell marker, but there is substantial evidence that demonstrates CD11c can also be expressed by certain subsets of macrophages.^46^ Further analysis demonstrated that approximately 60% to 100% of CD11c^+^ MHCII^+^ cells expressed F4/80 following BCG vaccination (Fig. 3D). F4/80 is typically defined as a macrophage marker, but it is important to note that most markers in our myeloid panel are not exclusive to a single cell type.^47^^,^^48^ Therefore, most of these cells are macrophages with potentially a smaller subset of dendritic cells present. Other combinations of myeloid cell markers were investigated but did not reveal any significant differences between unvaccinated and BCG vaccinated mice (Fig. S2). These findings demonstrated that there was an increase in the frequency of IL-27 producers that express MHC II as well as an increase in the level of expression following BCG vaccination, and that these cells were predominantly macrophages.

### A subpopulation of IL-27 producers in the lung that express MHC class II and Ly6G/C increases following BCG vaccination

**Figure 4.**
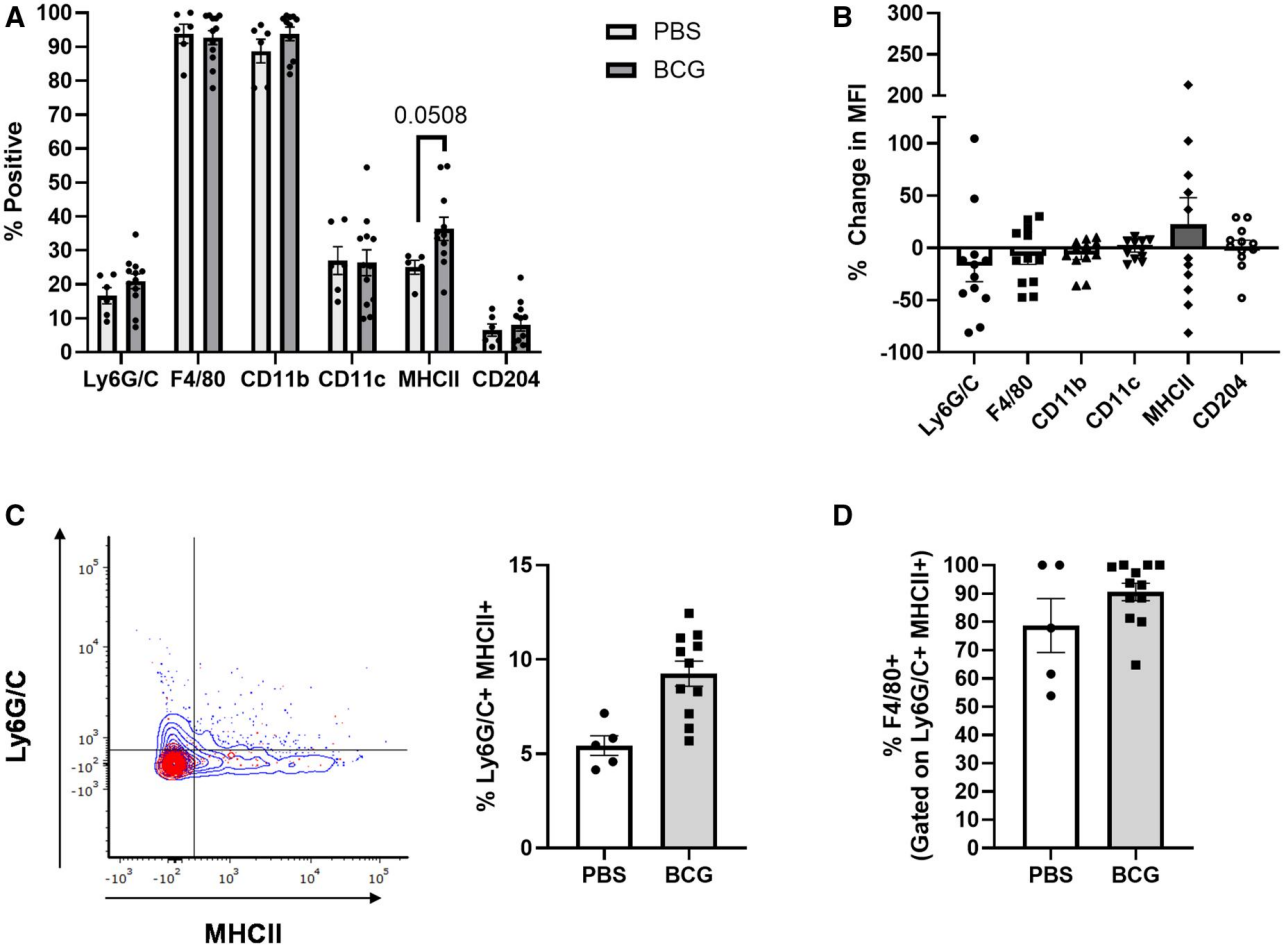
A subpopulation of IL-27 producers in the lung that express MHC class II and Gr-1 increases following BCG vaccination. At 5 weeks post-vaccination (WPV), lungs were collected from GFP mice. The lung cells were labeled with a live/dead stain and antibodies for CD3/CD19, CD45, Ly6G/C, F4/80, CD11b, CD11c, MHC II, and CD204. Flow cytometry was performed on the labeled cells. All data shown is gated on live CD3/CD19^-^GFP^+^ CD45^+^ cells. Data for individual markers are reported as (A) the mean percent of GFP^+^ cells ± SE also positive for the indicated markers and as (B) the mean percent change of median fluorescent intensity (MFI) of each marker between the PBS controls and BCG vaccinated mice ± SE. (C) A representative flow plot (red = control, blue = BCG vaccinated) and the corresponding mean Ly6G/C^+^MHCII^+^ percent of total cells ± SE is shown for both the control and BCG vaccinated groups. (D) The mean ± SE total frequency of Ly6G/C^+^MHCII^+^ cells that also express F4/80 is also shown. Data are representative of 4 independent experiments. Statistical analysis: (A, C, D) Unpaired *t* test, (G) Brown Forsythe and Welch ANOVA tests; *P < 0.05.

As with the spleen, we investigated the phenotype of IL-27 producers at 5 WPV in the lung due to the continuous increase in the abundance of IL-27 throughout the weeks following BCG vaccination. Lungs were harvested at 5 WPV and immunolabeled as described for the spleen and as shown in Fig. S1. Most IL-27 producers in the lung were F4/80^+^ and CD11b^+^ with a near significant increase in the frequency of MHC II expression following vaccination (Fig. 4A). An overall greater magnitude of MHC II expression was also evident by the MFI in some vaccinated animals (Fig. 4B). We also observed a significant increase in the percent of IL-27 producers that are both Ly6G/C^+^ and MHC II^+^ following vaccination (Fig. 4C). Ly6G/C expression is associated with granulocytes, monocytes, and macrophages.^49^^,^^50^ To determine whether the GFP^+^ Ly6G/C^+^ MHCII^+^ population represented macrophages or granulocytes, we analyzed the expression of F4/80 by these cells. Between 60% and 100% of these cells were F4/80^+^, suggesting a population of macrophages that expand nearly 2-fold following vaccination, with a smaller population of granulocytes potentially present (Fig. 4D). We analyzed other combinations of myeloid cell markers that did not reveal any significant differences between the unvaccinated and vaccinated groups (Fig. S3). Due to the significant increase in GFP^+^ cells at 3 WPV in the lung compared to other timepoints (Fig. 1E), we also phenotyped IL-27 producers at this timepoint. This analysis showed the populations to be relatively unchanged between the unvaccinated and vaccinated groups, but with modest increases in the level of MHC II and CD204 expressed per cell (Fig. S4). Collectively, these findings demonstrated an increased frequency of macrophage populations that produce IL-27 and increase expression of MHC II following BCG vaccination.

### scMultiome profiling of IL-27 producers reveals unique clusters of myeloid cells following BCG vaccination

**Figure 5.**
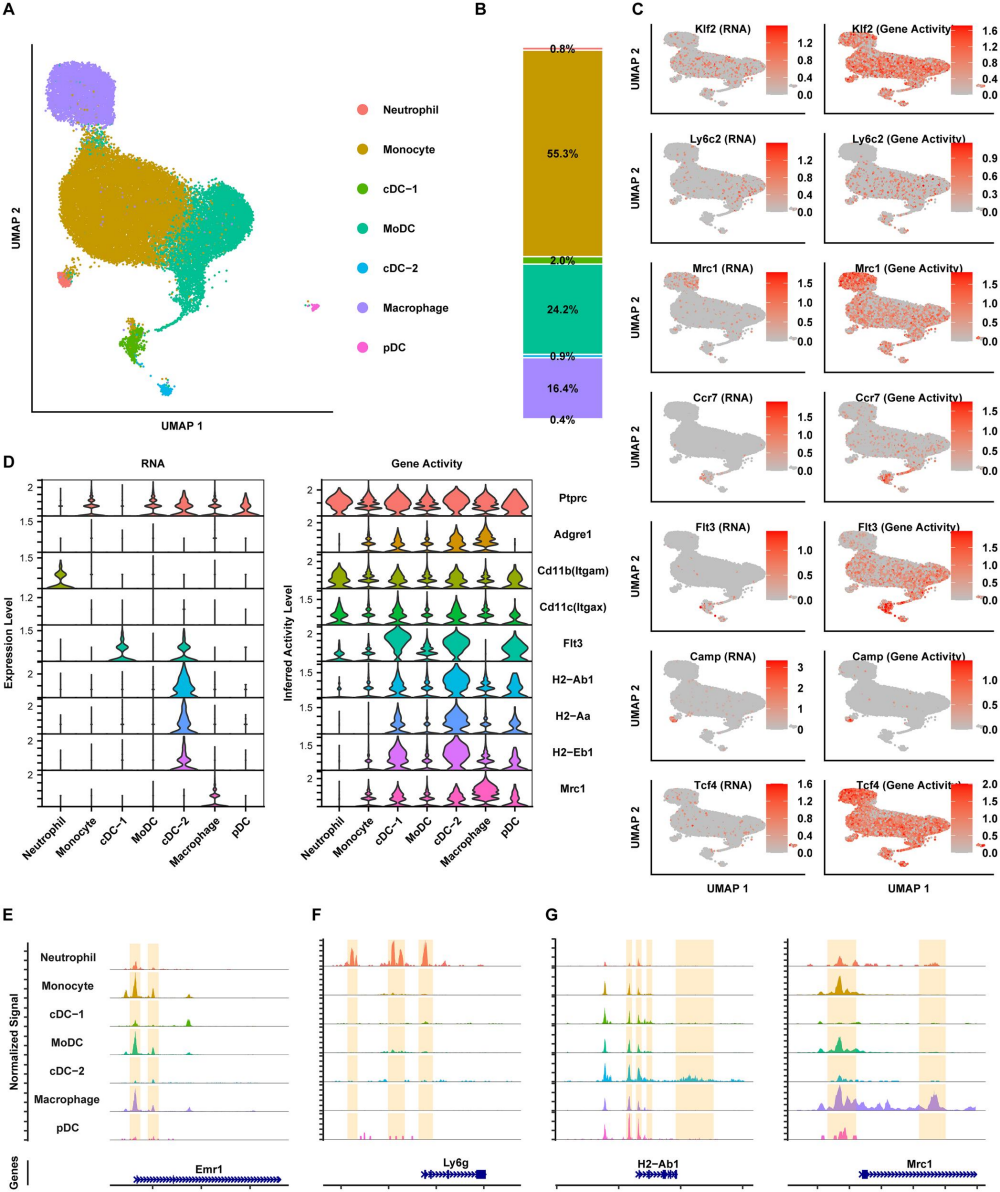
scMultiome profiling of IL-27 producers reveals unique clusters of myeloid cells. (A) Uniform manifold approximation and projection (UMAP) visualization of annotated clusters in the scMultiome dataset for IL-27 producers in the spleen. (B) Percent of each cell type from the scMultiome dataset. (C) UMAP visualization of gene expression from the RNA-Seq component and gene activity from the ATAC-seq component of the scMultiome dataset for markers genes used for cell type annotation. (D) Violin plots of RNA expression and gene activity based on RNA sequencing and ATAC-sequencing, respectively, for different myeloid genes. (E, F, G) Coverage plot showing the read distribution from the ATAC-seq component across representative myeloid genes with lineage selective chromatin openness at prompter regions or beyond promoter regions. Y-axis was set to the same scale for each gene to enable a direct comparison across cell types.

We selected the spleen as our primary tissue to profile at the single cell level due to the correlation between serum IL-27 and percent change in GFP MFI in the spleen (Fig. S2C). To complement the phenotypic analysis of IL-27 producers in the spleen and elucidate their functionality, we performed 10X scMultiome assays to profile paired gene expression (RNA-seq) and chromatin accessibility (ATAC-seq) at the single cell level. Spleens were harvested at 5 WPV from BCG-vaccinated GFP mice and the live GFP^+^ CD3/CD19^-^ negative cells were sorted using the gating scheme shown in Fig. S1. Integrated clustering analysis of the gene expression and ATAC-seq datasets revealed 7 clusters of IL-27-producing myeloid cells: neutrophils, monocytes, 2 populations of conventional dendritic cells (cDC-1 and cDC-2), monocyte-derived dendritic cells (MoDC), macrophages, and plasmacytoid dendritic cells (pDC) (Fig. 5A). The largest proportion of cells were annotated as monocytes, and this is supported by prior literature that identified a large population of undifferentiated monocytes in the spleen (Fig. 5B).^51^^,^^52^ Dendritic cell subsets comprised 27.5% of the total population of IL-27 producers, while macrophages were 16.4% of the total population (Fig. 5B). The annotations listed in Fig. 5A are based on the RNA expression and ATAC-seq inferred gene activity of cell-type specific marker genes such as *Klf2*, a transcription factor whose expression is high in monocytes but decreases upon differentiation (Fig. 5C).^53^  *Camp*, which encodes for the antimicrobial peptide cathelicidin, was used to annotate the neutrophil cluster as this protein is secreted by neutrophils, while *Ly6c2* expression helped to annotate the MoDC cluster (Fig. 5C).^54^^,^^55^ Additionally, the transcription factor *Tcf4* has been well-characterized for its role in pDC development, making it a suitable marker gene for this cluster (Fig. 5C).^56^^,^^57^ The annotations for the cDC populations are supported by the RNA expression and gene activity of *Flt3*, multiple MHC II alleles, and the chemokine receptor *Ccr7*, while macrophages are supported by the expression of *Mrc1* (Fig. 5C and D).^58–61^ It is important to note that in some instances, such as with *Flt3* and *Tcf4*, the chromatin was open across all clusters, but the RNA expression was exclusive to specific clusters (Fig. 5C and D). This suggests that other mechanisms, such as activation of enhancer elements, may be responsible for the observed gene expression.^62^ We also visualized the chromatin accessibility landscape of several myeloid cell genes (Fig. 5E–G). The chromatin for F4/80, encoded by *emr1*, was partially open in multiple clusters, with the greatest accessibility to the promoter in monocytic and macrophage clusters, and this was not surprising as flow cytometry data in Fig. 3 demonstrated that most IL-27 producers are F4/80^+^ (Fig. 5E).^63^ The chromatin for *Ly6G* was open at the promoter region in the neutrophil cluster, as expected based on high expression of Ly6G in neutrophils (Fig. 5F).^64^^,^^65^ Additionally, the promoters for *H2-Ab1* and *Mrc1* are partially open in most of the clusters, but downstream regulatory regions (relative transcription start sites) are open for only the cDC-2 and macrophage clusters, respectively (Fig. 5G). This suggests the RNA expression is regulated by these additional putative enhancer regions as expression of *H2-Ab1* is only observed in the cDC-2 cluster and expression of *Mrc1* is only observed in the macrophage cluster (Fig. 5D). This pattern of expression for *H2-Ab1* and *Mrc1* is expected based on prior literature.^59^^,^^66^

**Figure 6.**
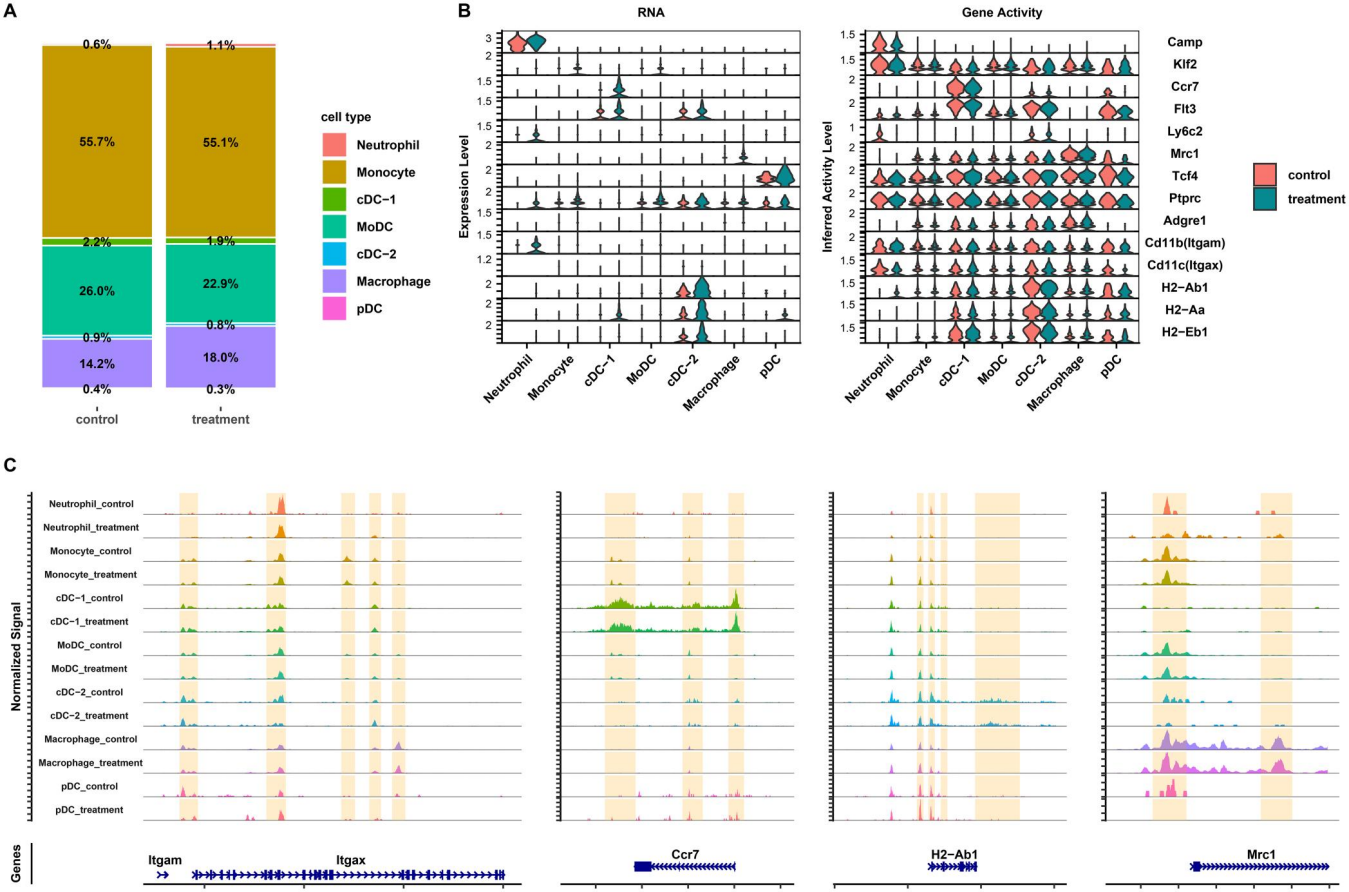
scMultiome profiling of IL-27 producers reveals unique characteristics of myeloid cells following BCG vaccination. (A) Percent of each cell type inferred from the scMultiome data of unvaccinated (control) versus BCG vaccinated (treatment) samples. (B) Violin plots of myeloid cell genes expression (left panel) and gene activity (right panel) based on the RNA-seq component and the ATAC-seq component of scMultiome, respectively, for vaccinated versus unvaccinated samples. (C) Coverage plot showing the read distribution from the ATAC-seq component across representative marker genes in control cells and treatment cells. *Y*-axis was set to the same scale for each gene to enable a direct comparison across cell types.

Evaluating differences between the unvaccinated and BCG vaccinated groups, the percent of cells in most clusters were similar between both groups; however, there was a modest decrease in MoDCs with a concomitant and comparable increase in macrophages following vaccination (Fig. 6A). The cluster-specific markers *Klf2* (monocyte), *Ccr7* (cDC-1), *Flt3* (cDC-2), *Ly6c2* (MoDC), *Mrc1* (macrophage), and *Tcf4* (pDC) all had increased gene expression following BCG vaccination (Fig. 6B, left panel). The increase in expression of *Mrc1* post-vaccination may be indicative of a subcluster of macrophages with heightened immunosuppressive activity, whereas increased *Ccr7* expression may represent mature cDCs with improved migration and/or recruitment to T cell zones (Fig. 6B).^59–61^ There was also increased expression of MHC class II genes post-vaccination in these cells (Fig. 6B). This supports the increase in MHC II expression by IL-27 producers in the spleen as shown by flow cytometry in Fig. 3A. In some instances, such as with *Flt3, Mrc1*, Tcf4, and MHC class II alleles, the gene activity inferred from chromatin accessibility at the promoter and gene body regions does not parallel the increase in gene expression following BCG vaccination, suggestive of additional enhancer or suppressor activity contributing to the transcription response to vaccination (Fig. 6B). We also inspected the genome browser tracks of different genes of interest to further examine differences between the two treatment groups and to understand the influence of chromatin accessibility on the observed gene expression. Specifically, we decided to look at CD11c (encoded by *Itgax*) because we observed an increase in CD11c^+^ MHC II^+^ cells post-vaccination in our flow cytometry data (Fig. 3C). We observed that the promoter for CD11c is open in most clusters (Fig. 6C). This is not unexpected as CD11c has been shown to be expressed by dendritic cells, plasmacytoid dendritic cells, macrophages, and monocytes.^46^ However, there are no differences in promoter openness between the unvaccinated and vaccinated groups. Similarly, promoter accessibility of *Ccr7*, *H2-Ab1*, and *Mrc1* was explored due to the increase in their gene expression post-vaccination. As expected, the promoters for *Ccr7* and *H2-Ab1* were most open in the dendritic cell clusters and the promoter for *Mrc1* was most open in the macrophage clusters, but there were no differences between the control and treatment groups for any of the three genes (Fig. 6C). All of these findings suggest the activation of additional BCG-driven regulatory regions for the observed gene expression activation of the marker genes.^62^

**Figure 7.**
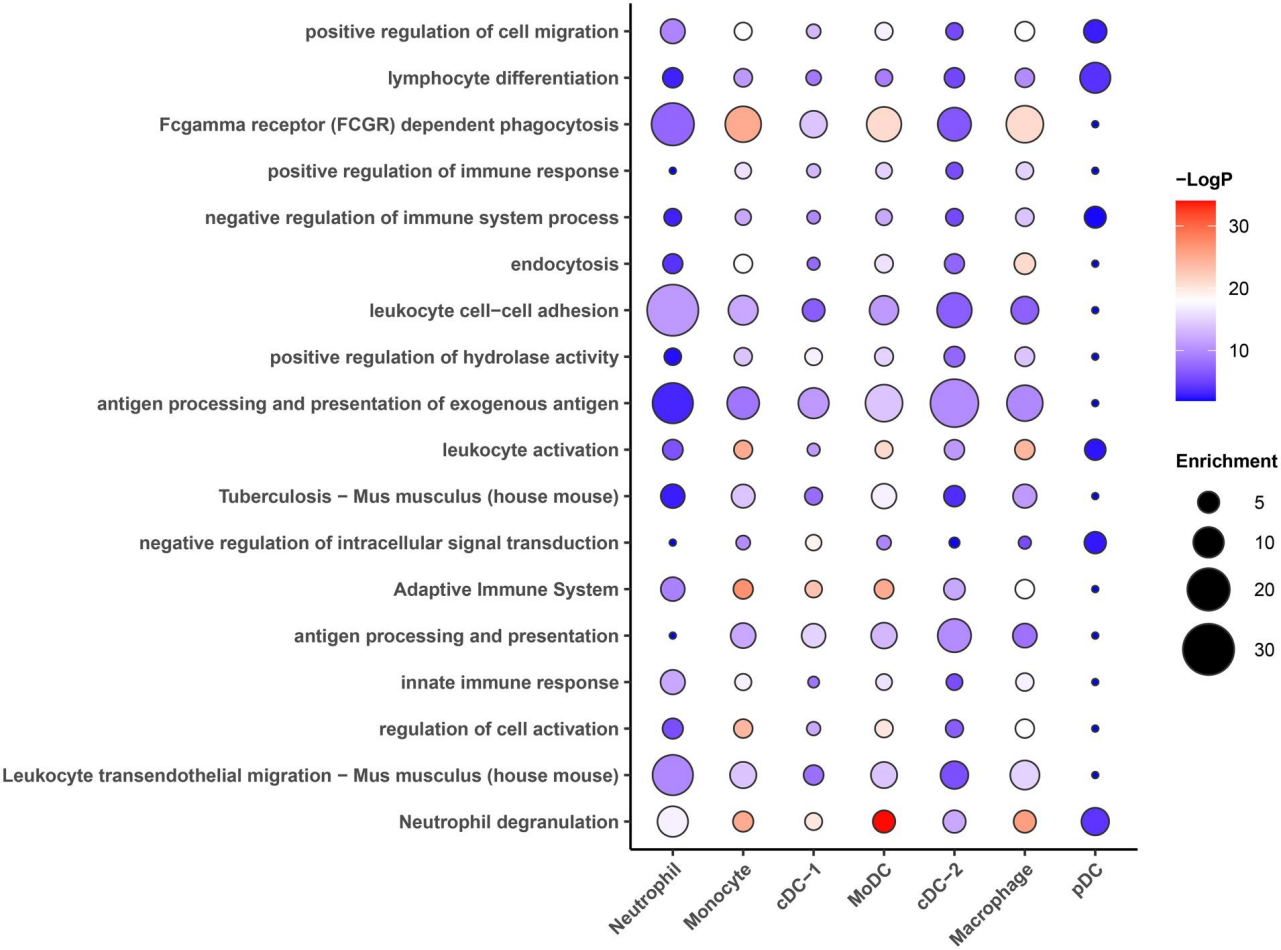
Gene ontology and pathway enrichment analysis demonstrates differential enrichment of gene sets following BCG vaccination. Data shown is for the gene ontology and pathway enrichment analysis of differentially expressed genes between the unvaccinated and vaccinated groups across the seven different clusters of IL-27 producers.

We then performed gene ontology (GO) and pathway enrichment analysis to further understand the functionality of IL-27 producing myeloid cells following BCG vaccination (Fig. 7). Leukocyte cell-cell adhesion and transendothelial cell migration gene sets were most enriched in the neutrophil cluster following BCG vaccination. We also observed that in the macrophage, monocyte, and MoDC clusters there was an enrichment in genes involved in phagocytosis and endocytosis, suggesting that cells in these clusters have more efficient uptake of antigen post-BCG vaccination. Genes in the “negative regulation of immune system process” were enriched in the monocyte and macrophage clusters, suggesting these cells may gain immunosuppressive functions following BCG vaccination. Additionally, there was enrichment of genes involved with antigen presentation and processing post-vaccination in most clusters, but particularly the cDC-2 cluster (Fig. 7). Similar findings were also evident for gene sets related with regulation of the adaptive immune system (Fig. 7). These findings suggest that the increased expression of MHC class II, as shown by our flow cytometry and the RNA-seq component of the scMultiome data in Figs. 3 and 6, is indicative of improved antigen processing and presentation by IL-27 producers following BCG vaccination. Overall, scMultiome data identified 7 clusters of heterogeneous IL-27-producing cells highlighted by subsets with functional profiles that suggest changes in antigen uptake, presentation, and cellular interactions following BCG vaccination.

### Persistent BCG is not responsible for the increase in IL-27 in the weeks following BCG vaccination

**Figure 8.**
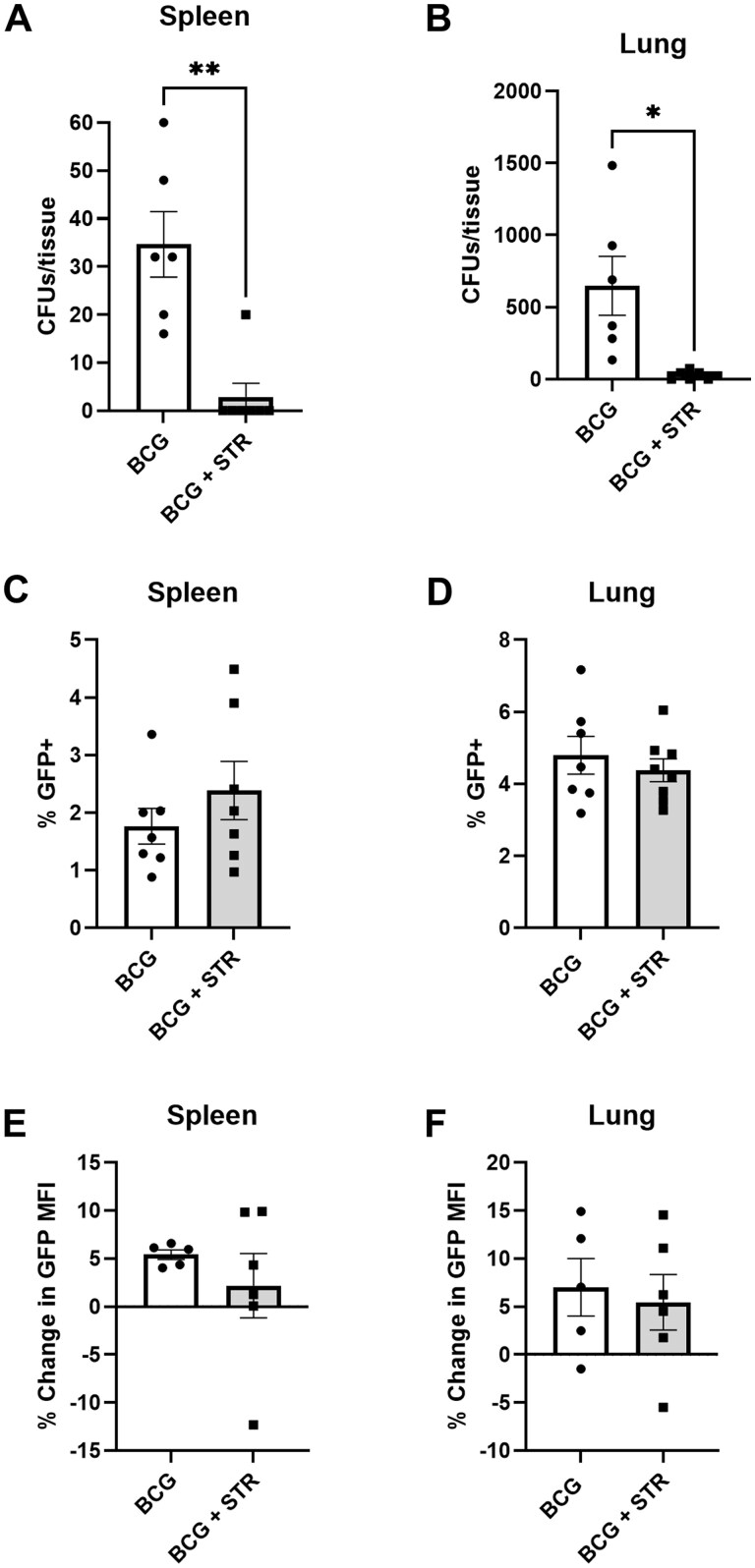
Persistent BCG is not responsible for the increase in IL-27 in the weeks following BCG vaccination. At 1 WPV, GFP mice were administered 150 mg/kg of streptomycin (STR) every other day for 2 weeks. Spleens were harvested following completion of streptomycin administration. Half of the spleen was homogenized, serially diluted and plated on 7H10 to determine bacterial burden. Splenocytes from the other half of the spleen were labeled with a live/dead stain and antibodies for CD3/CD19. Flow cytometry was performed on the labeled cells. Data are reported as (A, B) total colony forming units (CFUs) per tissue, the (C, D) mean percent of GFP+ cells ± SE, and the (E, F) the mean percent change of the median fluorescent intensity (MFI) of GFP expression ± SE relative to PBS controls. Data in (A–D) are representative of 3 independent experiments and data in (E, F) are representative of 2 independent experiments. Statistical analysis: (A, B, C, D) Unpaired *t* test; *P < 0.05, **P < 0.01.

There is a continual increase in the abundance of IL-27 produced per cell through 5 weeks post BCG vaccination as shown in Fig. 1 and in our previous work.^29^ We hypothesized that persistent BCG is driving the gradual increase in IL-27 over the weeks following BCG vaccination based on our findings and prior literature that demonstrates incomplete clearance of mycobacteria due to immune evasion mechanisms.^4^^,^^29^^,^^67–69^ To address this, BCG vaccinated mice were administered streptomycin every other day for 2 weeks, beginning at 1-week post-vaccination. Spleens and lungs were then harvested to determine bacterial burden and single cell suspensions of each tissue were labeled with a live dead stain and antibodies against CD3/CD19 for flow cytometry analysis. Streptomycin administration significantly decreased the bacterial burden in the spleen and lung (Fig. 8A and B). Unexpectedly, streptomycin treatment did not significantly change the percentage of GFP+ cells in the spleen or the lung (Fig. 8C and D). There also was no difference in the percent change of GFP MFI in either the spleen or lung following streptomycin treatment (Fig. 8E and F). Overall, these results indicate that while BCG persistent in the weeks following vaccination, they are not responsible for the gradual increase in IL-27 production.

## Discussion

In the present study, we investigated the cellular producers of IL-27 following BCG vaccination and their phenotypic and transcriptomic properties that may impact protection conferred by the vaccine. The source of IL-27 has been linked with downstream vaccine-induced responses in other models, but this has not been explored during BCG vaccination.^19^^,^^36^^,^^45^ IL-27 is a pleiotropic cytokine but its immunosuppressive functions, including impaired bacterial clearance and reduced lysosomal trafficking, are well documented.^14^^,^^19^^,^^29–32^ We have previously demonstrated that IL-27 interferes with the phagolysosomal pathway in macrophages via the downregulation of proteins such as V-ATPase.^31^^,^^32^ Additionally, we have shown that stimulation of cells with BCG or BCG vaccination further increases IL-27 levels.^14^^,^^29^^,^^32^^,^^35^ We believe these increased levels of IL-27 may further interfere with lysosomal activity, preventing efficient clearance of BCG and the formation of a protective immune response. This is suggested by our prior studies that demonstrated that in the absence of IL-27, mice vaccinated with BCG as neonates and challenged as adults clear *Mycobacterium tuberculosis* more effectively while generating a more diverse cytokine response.^29^^,^^35^ This underscores the importance of understanding IL-27 production in detail during the vaccination period. We hypothesized that unique subpopulations of IL-27 producers would arise following BCG vaccination and that their phenotype and function would be predicted to interfere with protective vaccine responses. To our knowledge, we are the first to phenotypically and transcriptionally profile IL-27 producers following BCG vaccination. We showed that BCG vaccination increases the frequency of IL-27-producing cells in both the spleen and lung following vaccination. We also identified IL-27 producers to be dominantly F4/80^+^ and CD11b^+^ with an increase in MHC II expression following vaccination in both tissues. Additionally, in the spleen there was an increase in CD11c^+^ MHC II^+^ IL-27 producers, whereas in the lung there was a significant increase in IL-27 producers that are Ly6G/C^+^ MHC II^+^ following vaccination. This highlights that IL-27 producers are not phenotypically uniform and unique subpopulations arise across tissues following BCG vaccination. Additionally, single-cell RNA sequencing identified these cells to primarily be monocytes with large populations of macrophages and dendritic cells also present. MHC II expression was increased post-vaccination in the dendritic cell clusters while *Mrc1* was increased in the macrophage cluster. Furthermore, some macrophage populations displayed heightened inflammatory responses following BCG vaccination, while others demonstrated immune suppressive functions. Taken together, this suggests that IL-27 producers display diverse immune functions post-vaccination.

Our prior work has demonstrated that BCG stimulation of human cells or vaccination of mice increases IL-27 production. More specifically, we have shown that the transcript levels for the p28 and EBI3 subunits increase when human monocyte-derived macrophages are infected with BCG.^32^ Similar findings were also observed in mice where bone marrow-derived dendritic cells produced significantly more IL-27 when stimulated with BCG.^29^ Mice vaccinated with BCG also experienced higher levels of circulating IL-27 compared to unvaccinated controls.^29^ The findings in the present study corroborate and extend these past results, as we demonstrated that BCG vaccination of IL-27p28eGFP (GFP) reporter mice increased IL-27 levels in serum alongside the frequency of IL-27 producing cells and their increasing magnitude of production in the spleen and lung. However, circulating IL-27 levels were surprisingly not detected until 3 weeks post-vaccination. We initially expected the bolus of bacteria to spike IL-27 levels early after vaccination. We also observed that the percent of producers peaks at 3 weeks post-vaccination in both tissues, while the per cell abundance of IL-27 produced increases throughout 5 weeks post-vaccination in the spleen and lung. We originally hypothesized that the delay in IL-27 detection until 3 weeks post-vaccination was caused by persistent BCG driving a continual increase in IL-27 levels. Previous studies have shown that both *Mycobacterium tuberculosis* and BCG are inefficiently cleared in humans and mice due to their ability to arrest phagosome maturation, along with other immune evasion capabilities.^4^^,^^67–69^ Our study showed that persistent BCG are not necessary for continued maintenance of IL-27-producing cells, as GFP expression does not decrease following clearance of BCG by streptomycin administration. An alternative explanation for the kinetics of IL-27 following BCG vaccination is trained immunity. Studies have shown that BCG can induce non-specific immunological memory in the innate immune system via epigenetic modifications, that can be detected up to 3 months after vaccination.^70–72^ For example, it has been shown that TNF-α production by PBMCs to Mtb, *S. aureus*, and *C. albicans* is higher from volunteers who have been BCG vaccinated.^70^ Other models have shown that trained immunity also increases the overall responsiveness of cells with both pro- and anti-inflammatory cytokines being increased upon restimulation,^73^ so it is possible that BCG vaccination trains cells to produce increased levels of IL-27 long-term and the persistent bacteria serves as a continual source of restimulation. However, our data only demonstrated minor differences in the chromatin availability of p28 and Ebi3 following BCG vaccination (data not shown). This does not account for common epigenetic signatures and future studies will be needed to fully determine if trained immunity contributes to the continual increase in IL-27 in the weeks following BCG vaccination.

Previous studies have demonstrated that different myeloid cell populations including macrophages, dendritic cells, and myeloid-derived suppressor cells are the dominant producers of IL-27.^16^^,^^19^^,^^20^ Additional evidence supports the notion that the cellular source of IL-27 can be both time- and context-dependent, and that the source can impact downstream immune responses.^19^^,^^36^^,^^45^ For example, a study demonstrated that following administration of certain vaccine adjuvants, IL-27 production from XCR1^+^ DCs and Ly6C^hi^ monocytes predicted the subsequent CD8^+^ T cell response.^36^ In our study, we found that approximately 80-90% of all IL-27 producers in the spleen and lung are F4/80^+^ and CD11b^+^. This was not surprising as many myeloid cells, including macrophages and granulocytes, can express F4/80 and CD11b.^47^^,^^48^^,^^74^^,^^75^ CD11b is also commonly expressed by subsets of dendritic cells.^46^ Our studies also revealed that the frequency of IL-27 producers that express MHC II increases following BCG vaccination in both tissues. Initially, this was unexpected since BCG has been shown to reduce MHC II expression under some conditions.^76^ However, it is important to highlight that we are studying IL-27 producers following BCG vaccination, not necessarily cells that contain BCG, although some IL-27 producers may. Alternatively, there are conflicting reports about the influence of IL-27 on MHC II expression with some studies demonstrating increased MHC II expression in the presence of IL-27 and others that show a decrease.^77–79^ These contrasting results are likely due to the pleiotropic nature of IL-27 where its effects can be context-dependent and either harmful or beneficial. Therefore, different study conditions, such as cell subset, stimulus, or disease model, can yield different outcomes that follow IL-27 signaling.

While IL-27 producers in the spleen and lung share many similarities, some differences also exist. In both tissues, there are small populations of MHC II^+^ cells that expand post-vaccination. In the spleen, a population of CD11c^+^MHCII^+^ cells expand, whereas in the lung there is a significant increase in Ly6G/C^+^MHCII^+^ IL-27 producers. While CD11c is typically recognized as a canonical dendritic cell marker, it is not specific to dendritic cells as macrophages and monocytes can also express CD11c.^46^^,^^80^ Additionally, F4/80 is primarily expressed by macrophages and monocytes but has been shown to also be expressed by certain dendritic cell subsets.^47^^,^^48^^,^^81^ Therefore, the CD11c^+^MHCII^+^ population in the spleen is likely a mixed population of macrophages and dendritic cells. In the lung, the Ly6G/C^+^MHCII^+^ population is also likely to be predominantly macrophages. The Ly6G/C antibody recognizes neutrophils, monocytes, and macrophages, but the co-expression of MHC II and F4/80 suggests this expanded population in the lung is macrophages.^47^^,^^48^^,^^50^^,^^81^ Overall, the subsets of IL-27 producers that acquire MHC II expression and expand following BCG vaccination differ phenotypically in the spleen versus the lung, but both may have improved antigen presentation capabilities based on their increased expression of MHC II post-vaccination.

Results from our scMultiome data analysis further supported an increase in MHC II expression by IL-27 producers following BCG vaccination. In the cDC-1, cDC-2, and pDC clusters, there is increased expression of *H2-Aa* following vaccination. It is important to note that most of the other clusters also express the MHC II alleles but at lower levels. Additionally, gene ontology enrichment analysis supports an enrichment in antigen processing and presentation pathways following BCG vaccination, especially in the cDC-2 cluster. We observed that most of the clusters also demonstrated enrichment in phagocytosis and hydrolase activity post-vaccination, further suggesting antigen uptake and processing.^82^ In addition, expression of *Ccr7* was increased in the cDC-1 cluster following BCG vaccination. CCR7 is a chemokine receptor that helps to guide dendritic cells to lymph nodes where they activate T cells.^60^^,^^61^ Therefore, increased *Ccr7* expression suggests that a population of cells in the cDC-1 cluster are mature dendritic cells homing to the lymph node post-vaccination. Despite well-documented immunosuppressive functions, IL-27 has also been shown to drive Th1 responses and this could explain the increase in antigen presentation observed post-vaccination.^17^ Alternatively, it has been shown that chronic infections, such as infection with BCG, can drive dysregulated adaptive immune responses.^4^^,^^83^ For example, persistent BCG antigen and continuous antigen presentation by IL-27 producers may be driving preferential generation of effector memory T cells over central memory T cells. Effector memory T cells are shorter-lived and preferential generation of them may negatively impact vaccine-induced responses.^4^^,^^84^ Overall, increased antigen presentation can have either a positive or negative influence on vaccine-induced responses and more studies would be necessary to fully elucidate the impact of these findings.

In addition to improved antigen presentation, our scMultiome data helped to further characterize other IL-27 producers. Gene expression analysis revealed an increase in the expression of *Mrc1* in the macrophage population following BCG vaccination. Increased *Mrc1* expression is associated with M2-like macrophages that have immunosuppressive phenotypes such as elevated production of IL-10.^59^^,^^85^ Gene ontology enrichment analysis identified “negative regulation of immune system process” as an enriched pathway in the macrophage cluster, further supporting a subpopulation of cells with an immunosuppressive phenotype post-vaccination. Maintenance of more regulatory macrophage populations may promote BCG persistence and prevent effective bacterial clearance upon Mtb exposure. As described above, bacterial persistence may drive dysregulated immune responses.^4^^,^^83^ However, gene ontology enrichment analysis also identified an enrichment in gene sets in the macrophage cluster that may contribute to bacterial clearance such as phagocytosis and antigen presentation. This suggests that the IL-27 producing macrophage population is heterogenous with subpopulations that may differ phenotypically and functionally.

In summary, these results highlight key IL-27 producers post-vaccination and demonstrate that these cells are a heterogenous population with diverse phenotypes and functions. Given the sustained level of IL-27 production from neonatal vaccination into adulthood, IL-27 is a significant immunological factor to consider in the vaccine-induced response for protection against TB. While we have shown that the net positive effect of global deletion of IL-27 signaling during BCG vaccination and Mtb challenge,^35^ this study identified that some populations of IL-27 producers likely provide beneficial roles during BCG vaccination. Future studies aimed at unraveling cell specific contributions will further enhance understanding of IL-27 regulation.

## Ethics statement

All procedures were approved by the West Virginia University Institutional Animal Care and Use Committee (IACUC) and conducted in accordance with the recommendations from the Guide for the Care and Use of Laboratory Animals by the National Research Council (NRC, 2011).

## Supplementary Material

vlaf003_Supplementary_Data

## Data Availability

The data underlying this article will be shared on reasonable request to the corresponding author.
