# Supplementary material for: IL-27 producers in a neonatal BCG vaccination model are a heterogenous population of myeloid cells that are diverse in phenotype and function
Source: Immunohorizons. 2025 Mar 6;9(4):vlaf003. doi: 10.1093/immhor/vlaf003 (PMC11884806; doi:10.1093/immhor/vlaf003)
Supplement: vlaf003_Supplementary_Data [file vlaf003_supplementary_data.pdf]

Supplemental Figures

Supplemental Figure 1

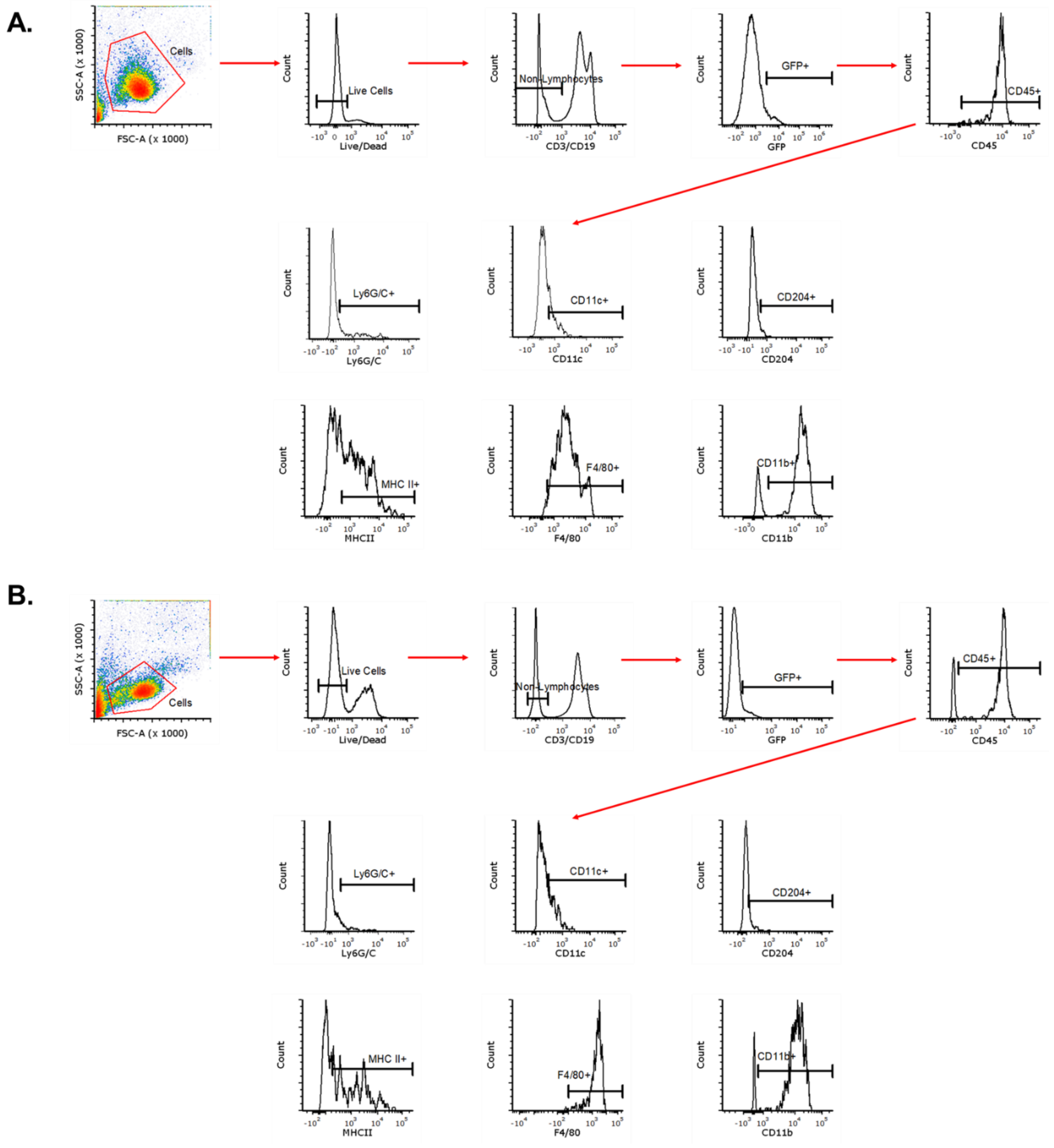

Supplemental Figure 1- Flow cytometry gating strategy for the phenotypic profiling of IL-27 producers in the (A) spleen and (B) lung.

Supplemental Figure 2

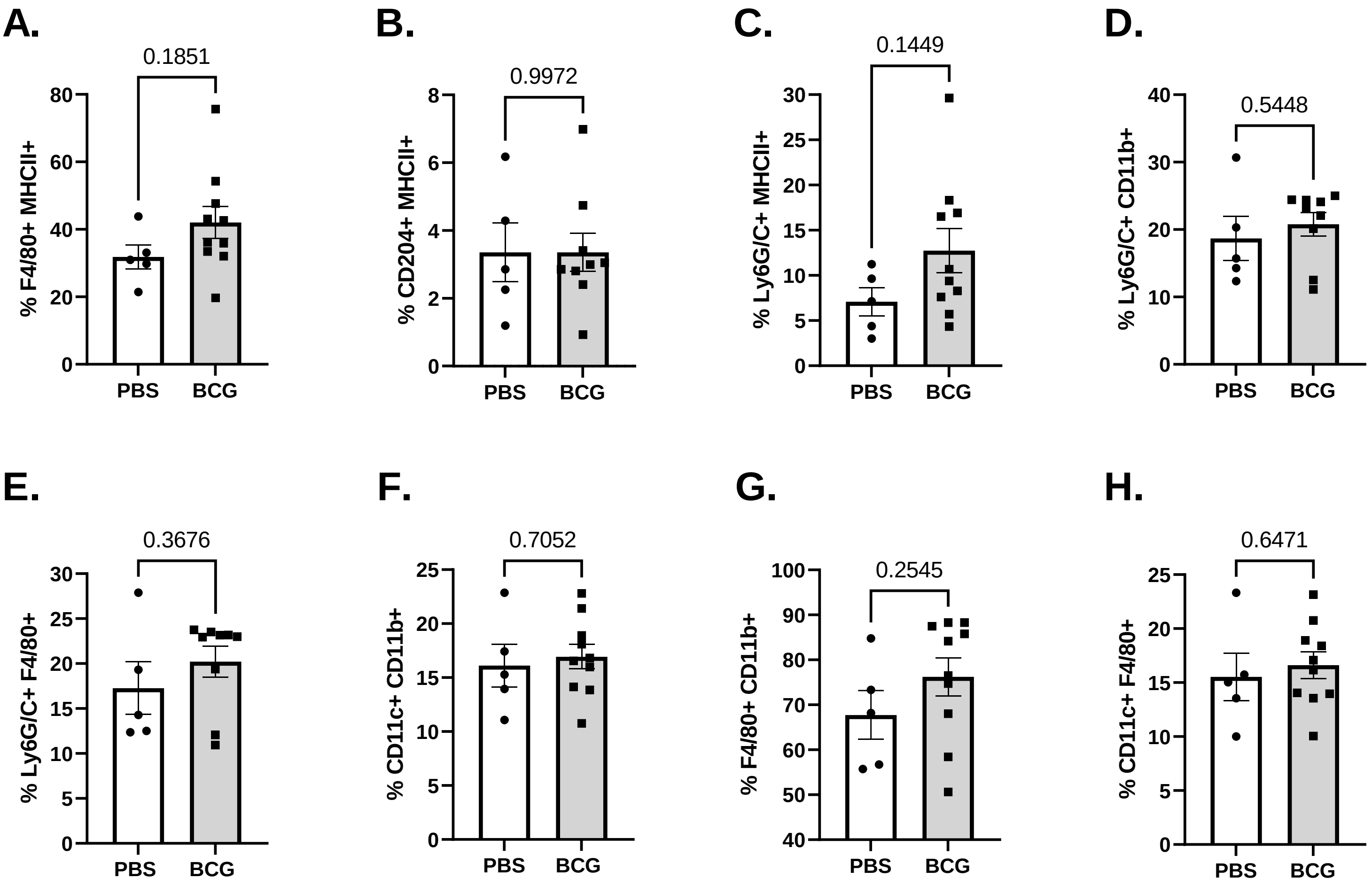

Supplemental Figure 2- Phenotype of IL-27 producers in the spleen at 5 weeks post BCG vaccination. At 5-weeks post-vaccination (WPV), spleens were collected from GFP mice. The splenocytes were labeled with a live/dead stain and antibodies for CD3/CD19, CD45, Ly6G/C, F4/80, CD11b, CD11c, MHC II, and CD204. Flow cytometry was performed on the labeled cells. All data shown is gated on live $CD3/CD19^{-}GFP^{+}CD45^{+}$ cells. Data for double positive populations are shown as the mean ± SE total frequency of (A) F4/80+ MHC-II+, (B) CD204+ MHC-II+, (C) $Ly6G/C^{+}$ $MHC\text{-}II^{+}$, (D) $Ly6G/C^{+}$ $CD11b^{+}$, (E) $Ly6G/C^{+}$ $F4/80^{+}$, (F) $CD11c^{+}$ $CD11b^{+}$, (G) $F4/80^{+}$ $CD11b^{+}$, and (H) $CD11c^{+}$ $F4/80^{+}$ populations in both the control and BCG vaccinated groups. Data are representative of 4 independent experiments. *Statistical analysis: Unpaired t test.*

Supplemental Figure 3

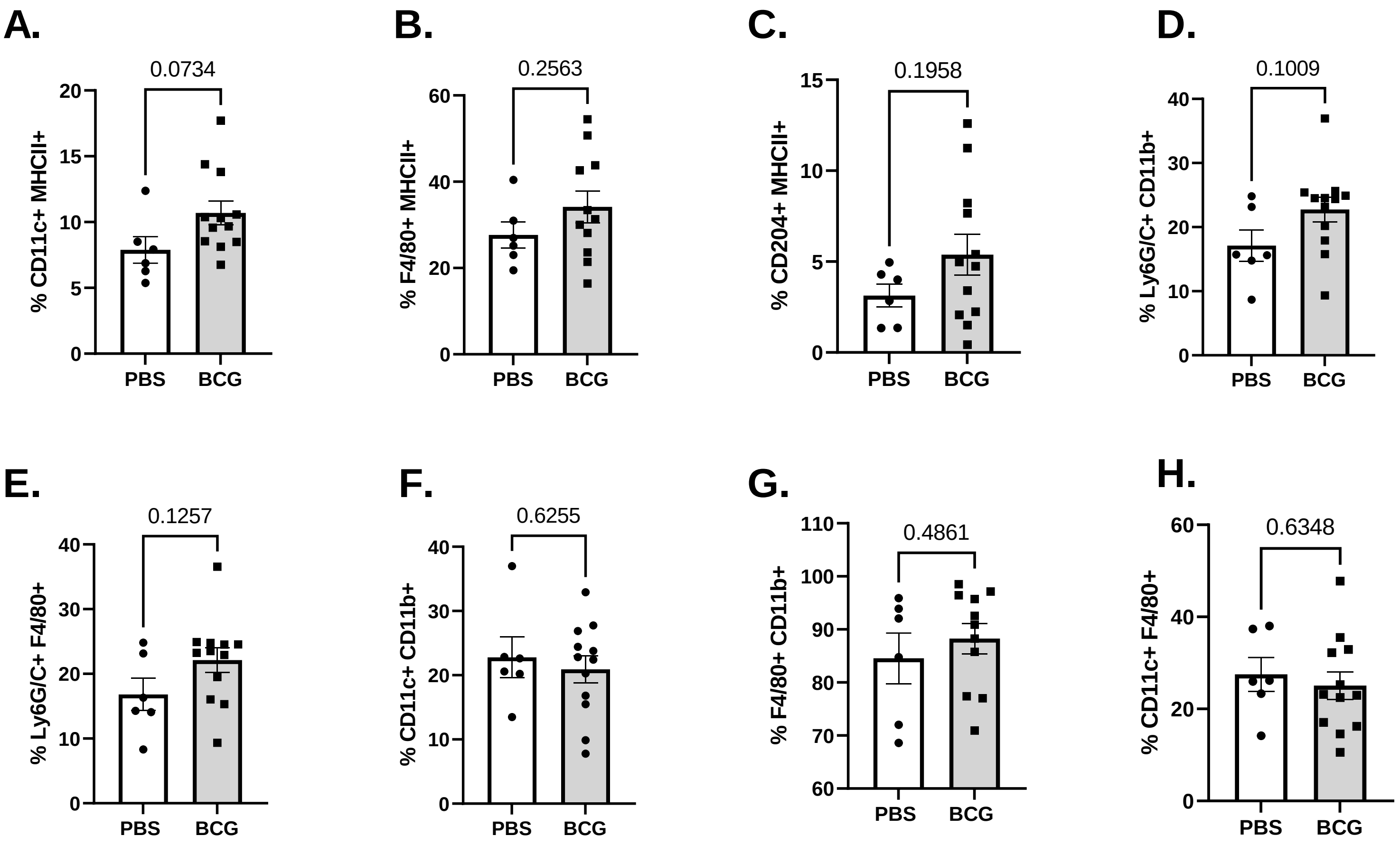

Supplemental Figure 3- Phenotype of IL-27 producers in the lung at 5 week post BCG vaccination. At 5-weeks post-vaccination (WPV), lungs were collected from GFP mice. The cells were labeled with a live/dead stain and antibodies for CD3/CD19, CD45, Ly6G/C, F4/80, CD11b, CD11c, MHC-II, and CD204. Flow cytometry was performed on the labeled cells. All data shown is gated on live $CD3/CD19^{-}GFP^{+}CD45^{+}$ cells. Data for double positive populations is shown as the mean ± SE total frequency of (A) $CD11c^{+}$ MHC II^{+}, (B) $F4/80^{+}$ MHC II^{+}, (C) $CD204^{+}$ MHC II^{+}, (D) $Ly6G/C^{+}$ $CD11b^{+}$, (E) $Ly6G/C^{+}$ $F4/80^{+}$, (F) $CD11c^{+}$ $CD11b^{+}$, (G) $F4/80^{+}$ $CD11b^{+}$, and (H) $CD11c^{+}$ $F4/80^{+}$ populations in both the control and BCG vaccinated groups. Data are representative of 4 independent experiments. *Statistical analysis: Unpaired t test.*

Supplemental Figure 4

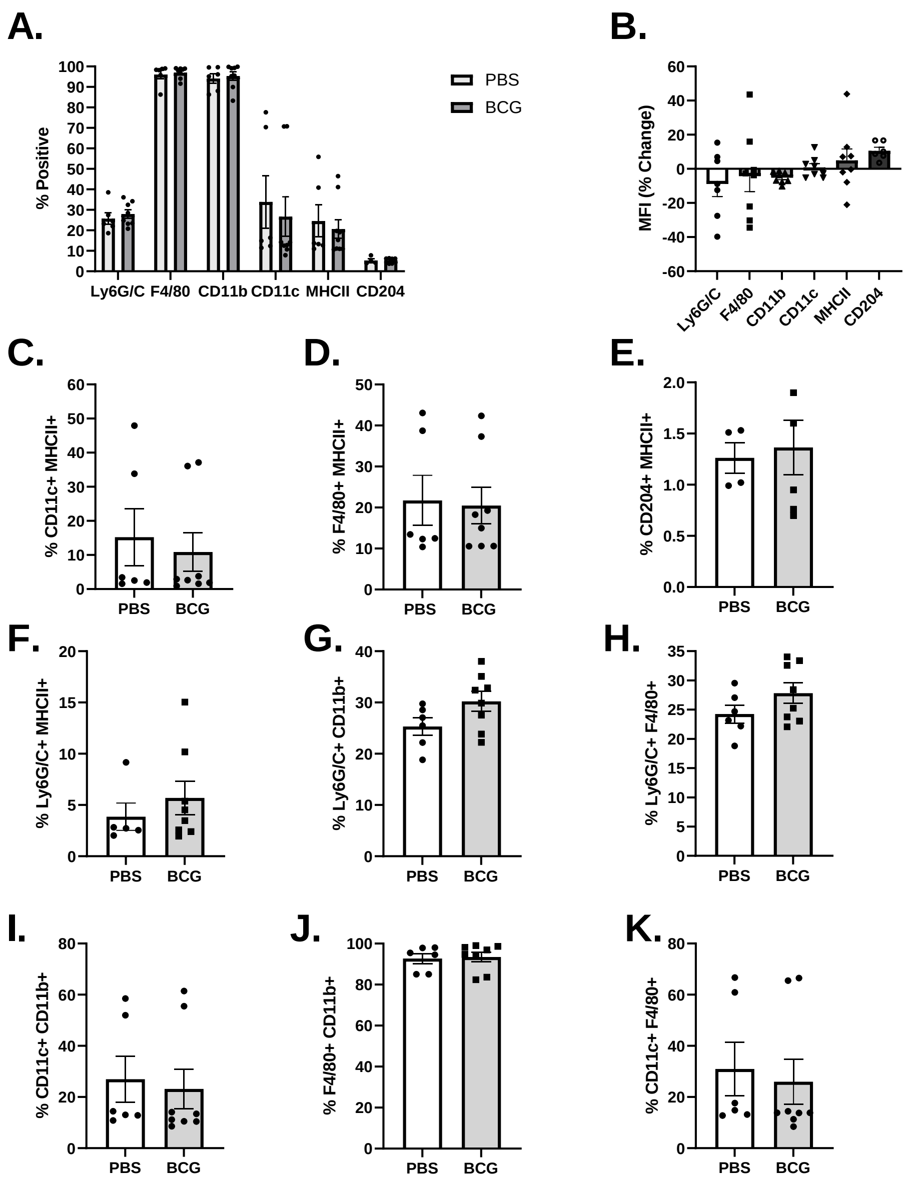

Supplemental Figure 4- Phenotype of IL-27 producers in the lung at 3 weeks post BCG vaccination. At 3-weeks post-vaccination (WPV), lungs were collected from GFP mice. The splenocytes were labeled with a live/dead stain and antibodies for CD3/CD19, CD45, Ly6G/C, F4/80, CD11b, CD11c, MHC II, and CD204. Flow cytometry was performed on the labeled cells. All data shown is gated on live CD3/CD19$^-$GFP$^+$CD45$^+$ cells. Data for individual markers is reported as (A) the mean percent of GFP$^+$ cells ± SE positive for the indicated markers and as (B) the mean percent change of median fluorescent intensity (MFI) of each marker between the PBS controls and BCG vaccinated mice ± SE. Data for double positive populations is shown as the mean ± SE total frequency of (C) CD11c$^+$ MHC II$^+$, (D) F4/80$^+$ MHC II$^+$, (E) CD204$^+$ MHC II$^+$, (F) Ly6G/C$^+$, MHC II$^+$, (G) Ly6G/C$^+$ CD11b$^+$, (H) Ly6G/C$^+$ F4/80$^+$, (I) CD11c$^+$ CD11b$^+$, (J) F4/80$^+$ CD11b$^+$, (K) CD11c$^+$ F4/80$^+$ populations in both the control and BCG vaccinated groups with representative flow plots. Data are representative of 3 independent experiments. *Statistical analysis: (A, C, D) Unpaired t test, (G) Brown Forsythe and Welch ANOVA tests.*
